# Alexithymia in the Narratization of Romantic Relationships: The Mediating Role of Fear of Intimacy

**DOI:** 10.3390/jcm13020404

**Published:** 2024-01-11

**Authors:** Elżbieta Zdankiewicz-Ścigała, Dawid Konrad Ścigała, Jerzy Trzebiński

**Affiliations:** 1 Faculty of Psychology, SWPS University, Chodakowska 19/33, 03-815 Warsaw, Poland; 2 Institute of Psychology, The Maria Grzegorzewska University, 02-353 Warsaw, Poland

**Keywords:** alexithymia, LIWC, narratization

## Abstract

Purpose: The purpose of the study was to verify the hypothesis concerning the relationship between alexithymia and selected indicators used to describe emotional events, specifically romantic relationships. Alexithymia, due to significant distortions in cognitive processing of emotional content, is demonstrated by poor recognition of emotions in oneself and others and, as a result, by deficits in empathy, avoidance of social relationships, and deficits in the ability to mentalize. Differences in narrations were tested by alexithymia levels (high vs. low) and the relation between specific narration features and individual alexithymia factors, i.e., difficulties in identifying emotions, difficulties in verbalising emotions, and externally oriented thinking. Method: A total of 356 people who had been in a romantic relationship for at least six months participated in the study. The TAS-20 was applied to measure alexithymia, and the FIS questionnaire was used to investigate anxiety in close relationships. Participants were asked to freely describe the romantic relationship they were in at that moment. The Linguistic Inquiry Word Count (LIWCLIWC2015 v1.6—unlimited duration academic licence) software was used for content analysis. The study was conducted online. Results: On the basis of the analyses conducted, high levels of alexithymia were found to be significantly associated with a lower total number of words used in narrative, a lower number relating to positive emotions, a lower number relating to causation and insight, and a higher number relating to negative emotions. Various results were obtained for individual dimensions of alexithymia in relation to the LIWC categories and the mediating role of fear of intimacy. For the difficulty identifying feelings (DIF), a significant mediating effect was observed only for words associated with negative emotions, whereas for the difficulty describing feelings (DDF), significant mediating effects were found for words relating to negative emotions and causality. In the case of externally oriented thinking (EOT), significant mediating effects were obtained for all analysed categories from LIWC.

## 1. Introduction

Alexithymia is conceptualised as a relatively stable feature of personality, characterised by deficits in experiencing, regulating, or verbalising emotions [1,2]. Individuals characterised by high levels of alexithymia present deficits in analysing their emotional conditions, manifested by externally oriented thinking that focuses on details and particulars related to an event, excluding insight into the individual’s internal states, which in turn hinders mentalization and results in a lack of imaginative life. They tend to create excessively general descriptions instead of recalling particular events, rich in details, emotions, etc., which significantly diminishes their narration. In addition, their descriptions do not have direct references to the self as the subject of specific emotional experiences. Alexithymia derives from a disconnection between conscious aspects of experience and perception at both mental and bodily levels. This results in difficulties in integrating thoughts, feelings, and experiences with consciousness and memory, negatively affecting awareness as well as emotion regulation and reflective functioning [3]. It may be due to the fact that alexithymia is associated with dysfunctional methods of regulating affect, such as conscious and deliberate avoidance of distressing desires, feelings, or experiences. It is furthermore associated with unconscious dissociation in cases of severe stress and difficulties in recalling distressing events or unpleasant experiences, as well as the use of intrusive defences such as projection or denial [4]. Such patterns of emotion modulation contribute to deficits in the process of mentalizing emotional experiences and limit awareness of desires and needs signalled by emotions [5]. Alexithymia is characterised not only by a problem with recognising and communicating emotions and outward-oriented thinking, but it is also associated with a disturbed process of affective self-regulation [6]. A great deal of research has been dedicated to verifying the hypothesis that interpersonal difficulties are evident in the romantic relationships of individuals with high levels of alexithymia [7,8,9,10,11,12].

## 2. Alexithymia and Fear of Intimacy in a Relationship

The difficulties described above that characterise people with high levels of alexithymia are largely rooted in the non-secure relationships in which children are raised [4]. Real-life experiences from infancy onwards influence the formation of intrapsychic internal working affective and cognitive representations (internal operational models of self, others, and self–others relationships), which constitute the basis for the exploitation and creation of complex emotion regulation strategies in a range of emotionally driven situations from childhood to adulthood [13]. Dysfunctional schemas form the basis for understanding oneself and others [14]. Particularly important for the development of alexithymia is the anxiety and avoidance style [15]. There are strong correlations between the development of alexithymia, insecure attachment, and emotional neglect (meta-analysis in [16]). It may be concluded that relationships in which people with alexithymia-indicating characteristics function from their childhood are due to negative emotional states experienced in the family. In the case of anxious and avoidant attachment, as many analyses have shown, families develop excessively rigid boundaries. Such boundaries between parents and children lead to isolation, especially in the area of emotional relationships. Such children get little warmth, care, and support when they are most needed. When emotional needs are not satisfied, self-sufficiency is forced as a result. At the cognitive level, pushing a child away brings an attitude representing the lack of interest in the world of an “other”, as well as significantly limited sharing of thoughts, own beliefs, etc. A deactivation of attachment and an attitude of defensive autonomy are typical for anxious and avoidant attachment [17,18]. This is sometimes referred to as hyper-individuation, i.e., the protection of the weak self through strong and rigid boundaries [19]. Failure to experience intimacy, support, interest, and warmth in childhood is conducive to developing shallow and superficial relationships. Maladaptive patterns, which arise during development, are not conducive to the evolution of basic interaction skills. Manifestations of the above include a low level of self-differentiation, the absence of mature intimacy, and the presence of hierarchical boundaries in the family system. Rigid boundaries, which protect the weak self, constitute a barrier to using feedback in a relationship without feeling threatened [13]. Failure to care about the relationship, failure to create shared activities, and failure to share features results in a strong sense of loneliness. Individuals with an anxiety and avoidance style of relationships suppress negative emotions [20,21]. This is especially true for emotions such as anxiety, anger, shame, and guilt, as these emotions may activate the attachment system. In addition, feelings of anxiety, anger, and shame may be perceived by these individuals as signals of weakness, which contradicts their self-image as strong and independent individuals. Furthermore, experiencing joy may be difficult for individuals with this attachment style, as it indicates closeness and involvement in a relationship. The aversion of people with an avoidant style to being involved in relations with others causes them to become less sensitive to other people’s signals [13]. Low levels of focus on emotional stimuli may also result from the reluctance to confront one’s inner self [4]. In studies on the recognition of facial expressions, the operation of defence mechanisms is demonstrated, among others, by diverting attention away from negative emotions and considering them as more neutral [22,23]. The consequences of such patterns include a deep fear of intimacy in a romantic relationship [9] and lower levels of self-differentiation [24]. Alexithymia, by protecting an individual against overstimulation or excess, becomes a cause for the development of deficits in cognitive and affective areas of emotional regulation [25]. By inhibiting the processes of identification and verbalization of emotions, it grossly distorts the basic function of language, i.e., interpersonal communication. It also hinders intrapersonal communication, i.e., understanding oneself. It may be predicted that these deficits negatively affect close relationships due to the absence of common meanings and accurate mutual understanding [26,27]. One study [24] verified the role of alexithymia in the context of the differentiation of the self in romantic relationships. A fear of intimacy and attachment styles in adulthood were included as mediators of such relationships. The analysis showed that alexithymia both directly and indirectly influenced the ability to differentiate oneself. The higher the level of alexithymia, the lower the level of differentiation of the self. This implies difficulty in emotion regulation in a relationship and the absence of balance between autonomy and building intimacy in a relationship. The fear of intimacy and anxious attachment styles in adulthood were found to be significant predictors [11]. Being in a relationship may also be difficult, as people with high alexithymia do not have the competence to understand others’ states of mind, which may cause anxiety. Therefore, we hypothesise that fear of intimacy could have a mediating role in the relationship between alexithymia and the narratization of romantic relationships.

## 3. Alexithymia and Dysfunctional Narratives

Few studies on the role of alexithymia in the narratization of autobiographical experiences suggest that individuals with high levels of alexithymia have difficulty recalling specific emotional episodes [28,29,30,31]. They tend to create excessively general descriptions instead of recalling specific episodes, rich in details, emotions, etc., which significantly weakens their narratives. Furthermore, their descriptions do not directly refer to the self as the subject of specific emotional episodes. Dimaggio et al. [32] proposed a division of dysfunctional forms of narrative into two basic categories, i.e., “narratives with integration deficit”, which comprise stories that are chaotic, shredded, and significantly impeding reception, and “impoverished narratives”—insufficiently articulated and varied to allow the narrator to locate themselves in the interpersonal relationships area. Such stories [33] are described as ‘barren’ and Polanyi [34] calls them ‘empty’—resembling, in their form, a report, a note, or a chronicle of events, without dramatising elements, emotion, or personal meaning. Impoverished narratives fall into two subcategories, i.e., production deficit narratives and alexithymic narratives. Alexithymic narratives, which constitute, in accordance with the typology proposed by Dimaggio et al. [32], a subcategory of impoverished narratives, are characterised by the absence of reference to affective states, difficulty in clearly describing the problem, poverty of imaginative life, excessive focus on specifics, disregard for the recipient’s perspective, absence of a sense of self-efficacy in important life matters, and absence of clear intention of the protagonist. They seem closer to externally oriented thinking than a narrative processing of experience over time. The authors indicate two critical moments where narrative impoverishment may take place. The first one is at the pre- and protonarrative level, where the absence of access to emotions or the ability to label them is linked to the difficulty in formulating intentions and goals that form the basis for future plans. Experience, deprived of an emotional core, reduces the narrative to a sequence of words and gestures from which it is difficult to infer the protagonist’s intentions. In this context, alexithymic self-narratives may thus be perceived as a deficit in narrative processing of experience, which manifests itself through the difficulty in developing coherent personal narratives [35]. Disturbances in the content and form of narratives may originate from disturbances at the level of unconscious procedural narrative. The other critical moment relates to acquiring dysfunctional schemas in the growth process and is associated with environmental factors such as non-secure attachment styles, early childhood trauma, or care neglect. The environment does not provide an individual with schemas, in the form of experiences or narratives, that animate and differentiate the world of their narratives [33]. Therefore, self-narratives are general or even ‘empty’ in terms of insights, emotions, and intentions to create imagined sequences of events resulting from intentions, meanings, and emotions.

Parker et al. [36] conducted a study in a sleep laboratory. Participants were requested to describe their dreams after waking up from the REM phase. They were further asked to rate the emotional valence of their dream experience. The authors demonstrated that there were no differences between the high and low alexithymic groups in the number of words that the participants used to describe their dreams. Similarly, no differences were found between the subjects in their assessment of the emotional value associated with the experience. Two independent assessors evaluated the subjects’ narratives in terms of the overall content of fantasy. Narratives developed by individuals with high levels of alexithymia were evaluated as significantly poorer on this dimension than those developed by individuals with low levels of alexithymia. These findings suggest that although alexithymia is not related to word production or emotional tone, it is negatively related to the production of abstract narratives.

## 4. Linguistic Indicators in Alexithymia

One of the methods that provides insight into the nature of language used by people with high levels of alexithymia is to analyse the content of linguistic productions, especially those concerning the narratization of emotions experienced in situations. This may be done, among others, by applying the linguistic inquiry word count (LIWC) [37]. Meganck et al. [31] examined a group of fifty mental inpatients. All of them complied with diagnostic criteria from Axis I in accordance with DSM-IV. The researchers used two tools to measure alexithymia, i.e., the Toronto alexithymia scale (TAS-20) questionnaire and the Toronto structured interview for alexithymia (TSIA). The LIWC software was used to analyse narratives. A clinical diagnostic interview was also conducted, from which a transcript was made, thus providing text material for analysis with the LIWC software. Despite disregarding the context of utterances, counting linguistic indicators makes it possible to analyse not only what an author says (content analysis) but also how they say it (style of expression). With LIWC, not only social aspects but also individual cognitive and emotional processes may be examined. The researchers focused on two categories from the LIWC dictionary relating to social processes, i.e., communication (e.g., talk, discuss, share, converse, argue) and references to other persons (e.g., you, everyone, colleagues, to them). Not only the frequency but also the complexity of wording (the number of different words within a category) were examined. A significant correlation was found between words referring to other people and the frequency of words in the communication category in patient speech and the interviewer’s reference to others. A negative correlation was found between the TAS-20 total score and word complexity, indicating lower complexity of vocabulary in the communication category in the speech of persons with high alexithymia; meanwhile, the TSIA total score was positively correlated with the number of words referring to other people. As for the TAS-20 subscales, the emotion identification dimension of the DIF had a negative correlation with the complexity of words referring to the communication category. As for the EOT (externally oriented thinking) subscale, in both the TAS-20 and TSIA, a negative correlation was found with communication word complexity, and a positive correlation was observed in the case of the ‘Other references to persons’ category. Further study by Vanheule et al. [38] focused not only on the social area but also on the cognitive functioning of persons with alexithymia. This time, a group of participants comprised 32 outpatients. They took part in a two-hour clinical interview, which was further transformed into a literal transcription, as in the study referred to above. Filling out the TAS-20 questionnaires was also part of the study. A hypothesis was tested relating to whether alexithymia would be associated with lower frequency and less variety of words in social processes (subcategories of the LIWC dictionary were as follows: communication, references to other people, family, friends, human beings), which would be expected to indicate social ‘disconnection’. The cognitive area was investigated using a cognitive processes category (LIWC subcategories included as follows: causality, insight, divergence, inhibition, uncertainty, and certainty). The researchers put into consideration whether the higher the level of alexithymia, the lower the frequency and variety of vocabulary in the cognitive processes category. The hypothesis was related to the observed cognitive deficits in emotion regulation and modulation. Data analysis showed that only the EOT (externally oriented thinking) subscale was significantly related to the LIWC categories and supported the hypothesis. The DIF dimension (difficulty identifying feelings) was positively correlated with the frequency of words from the cognitive processes category, which contradicts the hypothesis formulated. On the contrary, the DDF dimension (difficulty describing feelings) had no significant effect on the narratization process.

To look further into linguistic patterns used to describe affective experiences found in people with alexithymia Edwards et al. [29] conducted a study focusing on the analysis of content depending on pleasant and unpleasant experience descriptions. Taking into account previous research findings that report problems in emotional differentiation, especially in unpleasant contexts [39], writing tasks included a series of autobiographical open-ended questions (three descriptions each for positive and negative experiences), followed by subjects completing the positive and negative emotions scale (Polish: SUPIN). With this, the researchers obtained two emotion differentiation scores per person depending on the emotional marking of the experience. A total of 96 adults participated in the study, and the TAS-20 was applied to measure alexithymia. The tendency to adopt negative or positive language was analysed by deducting positive emotion scores from negative ones. Irrespective of the negative and positive context of experiences, alexithymia severity was significantly correlated with a tendency to use less positive vocabulary and a tendency to use negative language. In both pleasant and unpleasant descriptions of experiences, alexithymia severity level was significantly correlated with self-centeredness (more frequent use of the ‘I’ pronoun), which is not entirely consistent with the concept of low emotional self-awareness in people with alexithymia [29]. As far as the difficulty in differentiating emotions is concerned, this was positively correlated with the description of experiences irrespective of their affective label, suggesting that alexithymic individuals experience emotions more as a generalised affective state and are unable to use a subtle gradient of emotional valence and often of conflicting valence (feeling joy and sadness simultaneously for some important reason).

## 5. Purpose of This Study

So far, the research conducted has focused on memories from the past. Our research is exploratory in that it is the first study to analyse story-telling that takes place at present. It begins in the past, but it happens in the present. When analysing the results of various studies, including those described above, the role of alexithymia and its individual elements remains partially unclear. We hypothesise that people with high levels of alexithymia, due to deficits in emotional-relational functioning and in continuity with childhood attachment experiences, tend to avoid intimacy in their relationships. Specifically, it was assumed that alexithymia would be predictive of an overall lower number of words used in a narrative, a lower number of positive expressions, a higher number of negative expressions, and a lower number of terms relating to cognitive processes directly but also indirectly through fear of intimacy. Alexithymia factors would be to a different extent associated with the fear of intimacy, influencing the variation of expressions and their number in the romantic relationship narratization.

## 6. Description of the Study

### 6.1. Participants

The survey was conducted with a group of adults who had been in a romantic relationship for at least six months, although 53% of respondents reported that their relationship had been four years or longer. A total of 356 subjects participated in the survey, 80% of whom were women, and the average age of the respondents was M = 29.14; SD = 9.17. The survey was posted on Facebook and the USWPS website. It involved people who were actually in a romantic relationship. The relationships were not virtual. The subjects originated from the general population.

### 6.2. Research Tools

Toronto Alexithymia Scale TAS—20 PL (TAS-20) questionnaire in a Polish adaptation [40]. The questionnaire is used to measure the intensity of alexithymia and is a self-report. It enables calculating the overall level of alexithymia and three subscales relating to the following alexithymia dimensions: difficulty identifying feelings (DIF), difficulty describing feelings (DDF), and externally oriented thinking (EOT). The Toronto Alexithymia Scale comprises 20 items. A respondent addresses each by marking a response on a five-point Likert scale (1-totally disagree; 2-partially disagree; 3-I have no opinion; 4-partially agree; 5-totally agree). The overall scale ranges from 20 to 100 points. Alexithymia is treated as a dimension, the higher the score on the scale, the higher its level. Scores below 50 points are referred to as low levels of alexithymia, between 51 and 60 points, possible alexithymia, and scores above 61 points are referred to as high levels of alexithymia. The reliability of the scale expressed in Cronbach’s α in the study sample for the measure of general alexithymia is α = 0.85. In contrast, for the dimensions, Cronbach’s α is for DIF α = 0.81; DDF α = 0.78; and EOT α = 0.57, respectively. 

Fear-of-Intimacy Scale [41]. The FIS scale was developed to assess an individual’s fear of communicating thoughts and feelings in a close relationship. The FIS comprises 35 items, and responses are given on a five-point Likert scale with values ranging from 1 (completely disagree) to 5 (very much agree). The scale ranges from 35 to 175 points. A higher score indicates a greater fear of intimacy. Items are structured around three defining features: (a) content, communication of personal information; (b) emotional valence, strong feelings about the personal information exchanged; (c) sensitivity, high respect for the other person’s intimacy. The Cronbach’s alpha reliability coefficient for the sample is α = 0.95.

Analysis of romantic relationship description. A final stage of the study was the respondents’ description of their romantic relationship. They were requested to describe their romantic relationships as freely as possible, without paying attention to errors or writing style. The written content was then subjected to linguistic analysis. This was conducted using the Linguistic Inquiry Word Count (LIWC) software designed by Pennebaker [37]. The categories are divided into four dimensions: basic parts of speech (e.g., pronouns, numerals, prepositions), psychological processes (e.g., affective processes—positive emotions and negative emotions, cognitive processes—insight, causality, certainty), categories relating to relativity, connections between objects (e.g., motion, time, space), and categories relating to everyday matters (e.g., work, home, food, music) [37]. Each word or phrase may be assigned to one or more categories, which often have a hierarchical structure. A Polish adaptation of the software [42] was used for the analysis. The programme analyses a text by counting the number of words in it that belong to selected linguistic and psychological categories. This provides information about the frequency of words from specific categories. The LIWC makes it possible to both identify words that carry content and those that provide speech coherence. Each word in the analysed text is referred to and compared to a specific dictionary. Before the analyses, the texts had been checked and corrected in terms of missing letters and spelling errors, as these could significantly affect the quality of the analysis. To analyse narratives about romantic relationships, categories relating to psychological processes were used, i.e., affective processes and cognitive processes.

### 6.3. Procedure

Before the study, approval from the Ethics Committee of the USWPS Department of Psychology (No. 61/2021) was obtained. The survey was conducted online. Two identical questionnaires were developed—one in Qualtrics and the other in Google Forms. After giving consent to participate in the survey, participants gave information on their gender, age, country of residence, and duration of their romantic relationship. As regards the length of romantic relationships, they could choose from the following timeframes, i.e., six months–one year, one year–two years, two years–three years, or over three years. They were further asked to fill out two questionnaires in the following order: TAS-20, FIS. In the last stage, the subjects were requested to freely describe the romantic relationships they were currently in. The instructions for writing a response were as follows: “*Describe the beginning of your relationship and its subsequent stages. Try to describe everything that was important in it. Also describe what is happening in your relationship now—where you are in the relationship at the moment. Write freely, you don’t need to care about style or worry about mistakes. Treat it as writing for yourself. Don’t care about the size of the window below. You can enlarge it with the slider on the right side of the screen, so your description may be as long as you want. If you have already finished writing, take some more time to read the entire text. You may add to or revise the description to reflect what has been, is, and may be happening in your relationship*”.

## 7. Results

### 7.1. Analytical Strategy

The analysis of results was commenced by checking relationships between variables from the self-report scales (alexithymia and fear of intimacy) with dimensions relating to narratization (quantity of words, affective processes, and cognitive processes) with the Pearson’s r linear correlation coefficient. The following step was to verify differences using one-way analyses of variance (ANOVA) in narratization between those who scored at least 51 points on the alexithymia dimension (above the 75th percentile) and those with low scores, i.e., those who scored up to the 25th percentile. The level of 51 points is important due to the fulfilment of a criterion of probable alexithymia, according to Taylor and Bagby. The final stage in the data analysis was to verify the mediating role of fear of intimacy in the relationship between alexithymia and the narratization dimensions (total words, total terms for positive and negative emotions, and causality out of cognitive processes). The mediation method enables to verify the impact of the mediator and resulting variables while partially removing the relationship between the predictor and the resulting variables; thus, the mediation analysis allows to verify assumptions based on theory [43], and therefore it was used to analyse the results. In the analysis, a non-standard window was applied for SPSS Process Macro by A.F. Hayes model 4 [44]. A customary threshold of *p* < 0.05 was adopted to constitute the significance level, although to achieve a more accurate level of estimation for assessing indirect effects, confidence intervals (CI) based on the bootstrapping method with a sampling of 10,000 were tested. Bearing in mind the results obtained by other researchers regarding the analysis of autobiographical narratives in which the role of particular factors of alexithymia was examined, we decided to follow this analytical path and investigate individual factors in the context of fear of intimacy and the language used to describe romantic relationships.

### 7.2. Descriptive Statistics and Correlations

Out of the data in Table 1, it is worth noting a strong positive correlation between alexithymia and fear of intimacy and between alexithymia and the total number of terms for negative emotions in the description of a romantic relationship. In addition, it is also noteworthy that there is a negative correlation between the level of alexithymia and the total number of words in the narrative and the number of terms for positive emotions in the narrative and alexithymia. The results obtained support the study hypotheses adopted.

In the subsequent step of the analyses, individuals with high (N = 89) alexithymia, i.e., those who meet the alexithymia criterion according to Parker et al. [2], and low (N = 87) alexithymia levels were selected from the entire study group in order to investigate whether there are any differences in the level of the analysed variables depending on the level of alexithymia. It was found in an ANOVA analysis of variance that those with high alexithymia levels have a significantly higher level of fear of intimacy in close relationships, a significantly higher rate of words relating to negative emotions presented in the narrative, and a lower rate of words relating to positive emotions (Table 2). Significant differences between groups also refer to a number of terms relating to describing cognitive processes and specifically to causality. There are significantly fewer of these in the group with a high alexithymia level. 

**Figure 1:**
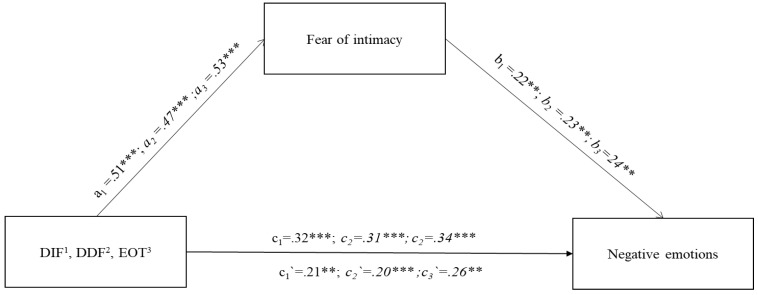
Path diagram of the three indirect effects of alexithymia subscales (DIF, DDF, EOT) on LIWC subscale negative emotions. Note. The figure presents standardised coefficients. All mediation models are statistically significant. The figure presents three models for the three dimensions of alexithymia, i.e.,: 1—DIF (a_1_,b_1_,c_1_/c_1_′); a_1_ = relation between DIF and Mediator (Fear of intimacy); b_1_ = relation between Mediator (Fear of intimacy) and LIWC Negative emotions; c_1_ = direct effect of DIF on LIWC Negative Emotions; c_1_′ = indirect effect of DIF on LIWC Negative Emotions through Mediator (Fear of intimacy), 2—DDF (a_2_,b_2_,c_2_/c_2_′); a_2_ = relation between DDF and Mediator (Fear of intimacy); b_2_ = relation between Mediator (Fear of intimacy) and LIWC Negative emotions; c_2_ = direct effect of DDF on LIWC Negative Emotions; c_2_′ = indirect effect of DDF on LIWC Negative Emotions through Mediator (Fear of intimacy), 3—EOT (a_3_,b_3_,c_3_/c_3_′) a_3_ = relation between EOT and Mediator (Fear of intimacy); b_3_ = relation between Mediator (Fear of intimacy) and LIWC Negative emotions; c_3_ = direct effect of EOT on LIWC Negative Emotions; c_3_′ = indirect effect of EOT on LIWC Negative Emotions through Mediator (Fear of intimacy), ** *p* < 0.01; *** *p* < 0.001.

The following stage of the analyses referred to investigating a direct relationship between alexithymia and the use of negative emotion words, positive emotion words, and causality words in narrative, taking into account an indirect effect of fear of intimacy. The first model tested verified the mediating role of fear of intimacy on the relationship between the three dimensions of alexithymia (DIF, DDF, and EOT) and the total negative emotion terms used in the narrative (Figure 1). For all three dimensions, the models obtained fit the data well. DIF F(2.243) = 19.71; *p <* 0.001; explaining 37.4% of the variability in negative emotions; DDF F(2.243) = 19.41; *p <* 0.001; explaining 37.1% of the variability in negative emotions; EOT F(2.243) = 24.43; *p <* 0.001; explaining up to 41.0% of the variability in negative emotions. A direct relationship for each scale with the total negative emotions was at a similar level, respectively: DIF c = 0.32; *p <* 0.001; DDF c = 0.31; *p <* 0.001; and EOT c = 0.34; *p <* 0.001.

The relationship of the independent variables (DIF, DDF, and EOT) with the mediator (FIS) was strongest for the EOT dimension (a_3_ = 0.53; *p* < 0.001); for other dimensions, it was also significant and was DIF a_1_ = 0.51; *p* < 0.001; and DDF a_2_ = 0.47; *p <* 0.001. The next step involved verifying the mediator’s (FIS) relationship with the total words for negative emotions. The relationship of this variable with the total number of negative emotions used in the narrative was also found to be significant. DIF b_1_ = 0.22; *p* < 0.001; DDF b_2_ = 0.23; *p* < 0.001; EOT b_3_ = 0.24; *p* < 0.001.

After introducing the mediator into the model, the relationship between the alexithymia dimensions and the number of words describing negative emotions decreased for each relationship. DIF c_1_′ = 0.21; *p* < 0.001; DDF c_2_′ = 0.20; *p* < 0.01; EOT c_3_′ = 0.26; *p* < 0.01, but in each case, the relationship remained significant. Summarising all mediation models was found to be partial and significant, as indicated also by significant indirect effects for each model based on the bootstrapping method with 10,000 sampling points: DIF (b = 0.11, 95% CI [0.04; 0.19]); DDF (b = 0.11, 95% CI [0.04; 0.18]); and EOT (b = 0.08, 95% CI [0.04; 0.12]).

**Figure 2:**
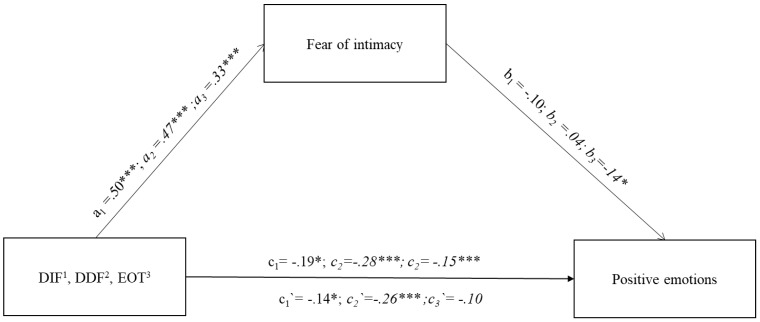
Path diagram of the three indirect effects of alexithymia subscales (DIF, DDF, EOT) on LIWC subscale positive emotions. Note. The figure presents standardised coefficients. The figure presents three models for the three dimensions of alexithymia, i.e.,: Only the mediation model for EOT is statistically significant. 1—DIF (a_1_,b_1_,c_1_/c_1_′); a_1_ = relation between DIF and Mediator (Fear of intimacy); b_1_ = relation between Mediator (Fear of intimacy) and LIWC Positive emotions; c_1_ = direct effect of DIF on LIWC Positive emotions; c_1_′ = indirect effect of DIF on LIWC Positive emotions through Mediator (Fear of intimacy), 2—DDF (a_2_,b_2_,c_2_/c_2_′); a_2_ = relation between DDF and Mediator (Fear of intimacy); b_2_ = relation between Mediator (Fear of intimacy) and LIWC Positive emotions; c_2_ = direct effect of DDF on LIWC Positive emotions; c_2_′ = indirect effect of DDF on LIWC Positive emotions through Mediator (Fear of intimacy), 3—EOT (a_3_,b_3_,c_3_/c_3_′) a_3_ = relation between EOT and Mediator (Fear of intimacy); b_3_ = relation between Mediator (Fear of intimacy) and LIWC Positive emotions; c_3_ = direct effect of EOT on LIWC Positive emotions; c_3_′ = indirect effect of EOT on LIWC Positive emotions through Mediator (Fear of intimacy), * *p* < 0.05; *** *p* < 0.001.

The second mediation model investigated the mediating role of fear of intimacy on the relationship between the three dimensions of alexithymia (DIF, DDF, and EOT) and the total terms referring to positive emotions used in the narrative (Figure 2). For all three dimensions, the models obtained fit the data well. DIF F(2.243) = 5.64; *p* < 0.05; explaining 21% of the variability in positive emotions; DDF F(2.243) = 10.88; *p* < 0.001; explaining 28.7% of the variability in positive emotions; EOT F(2.243) = 4.97; *p* < 0.05; explaining 19.8% of the variability in positive emotions. The direct relationship between each scale and total positive emotions was observed at different levels. Respectively, DIF c_1_ = −0.19; *p* < 0.001; DDF c_2_ = −0.28; *p* < 0.001; and EOT c_3_ = −0.15; *p* < 0.05. The relationship of the independent variables (DIF, DDF, and EOT) with the mediator (FIS) was significant for each dimension (DIF a_1_ = 0.50; *p* < 0.001; DDF a_2_ = 0.47; *p* < 0.001; EOT a_3_ = 0.33; *p* < 0.001). When a mediator (FIS) was introduced into the model, the relationship of this variable with the total words used to describe positive emotions proved to be significant only for externally oriented thinking (EOT b_3_ = −0.14; *p* < 0.001). Therefore, only the mediation model for externally oriented thinking was found to be significant, and it is a full mediation, because after the introduction of the mediator (FIS), the relationship of externally oriented thinking with the total words used to describe positive emotions decreased to a non-significant level. EOT c_3_′ = −0.10; *p* > 0.05.

The existence of a significant mediation model is confirmed by an indirect effect score based on the bootstrapping method with 10,000 samples (b = −0.04, 95% CI [−0.09; −0.01]).

**Figure 3:**
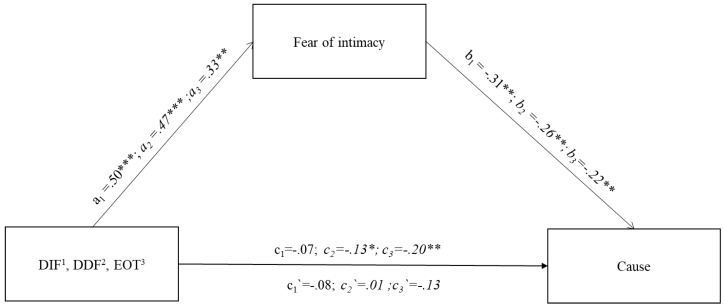
Path diagram of the three indirect effects of alexithymia subscales (DIF, DDF, EOT) on LIWC subscale cause. Note. The figure presents standardised coefficients. The figure presents three models for the three dimensions of alexithymia, i.e., mediation models for DDF and EOT are statistically significant. 1—DIF(a_1_,b_1_,c_1_/c_1_′); a_1_ = relation between DIF and Mediator(Fear of intimacy); b_1_ = relation between Mediator(Fear of intimacy) and LIWC Cause; c_1_ = direct effect of DIF on LIWC Cause; c_1_′ = indirect effect of DIF on LIWC Cause through Mediator(Fear of intimacy), 2—DDF (a_2_,b_2_,c_2_/c_2_′); a_2_ = relation between DDF and Mediator(Fear of intimacy); b_2_ = relation between Mediator(Fear of intimacy) and LIWC Cause; c_2_ = direct effect of DDF on LIWC Cause; c_2_′ = indirect effect of DDF on LIWC Cause through Mediator(Fear of intimacy), 3—EOT (a_3_,b_3_,c_3_/c_3_′) a_3_ = relation between EOT and Mediator(Fear of intimacy); b_3_ = relation between Mediator(Fear of intimacy) and LIWC Cause; c_3_ = direct effect of EOT on LIWC Cause; c_3_′ = indirect effect of EOT on LIWC Cause through Mediator(Fear of intimacy), * *p* < 0.05; ** *p* < 0.01; *** *p* < 0.001.

The third mediation model verified the mediating role of fear of intimacy on the relationship between the three dimensions of alexithymia (DIF, DDF, and EOT) and terms related to causality in the narrative (Figure 3). For all three dimensions, the models obtained fit the data well: DIF F(2.243) = 10.03; *p* < 0.001; explaining 27.6% of the variability in causality; DDF F(2.243) = 9.27; *p* < 0.01; explaining 26.6% of the variability in causality; EOT F(2.243) = 11.34; *p* < 0.001; explaining 29.2% of the variability in causality.

The direct relationship was different for particular dimensions, i.e., DIF c_1_ = −0.07; *p* > 0.05; DDF c_2_ = −0.13; *p* < 0.05; and EOT c_3_ = −0.20; *p* < 0.001, respectively, which means in this case that the mediation model for the dimension of difficulty identifying emotions (DIF) cannot be fully verified because there is no direct relationship of the mentioned dimension with causality. The relationship of the independent variables (DIF, DDF, and EOT) with the mediator (FIS) was significant for each dimension (DIF a_1_ = 0.50; *p* < 0.001; DDF a_2_ = 0.47; *p* < 0.001; EOT a_3_ = 0.33; *p* < 0.001). When a mediator (FIS) was introduced into the model, the relationship of this variable with causality terms was also found to be significant (DIF b_1_ = −0.31; *p* < 0.001; DDF b_2_ = −0.26; *p* < 0.001; EOT b_3_ = −0.22; *p* < 0.001). However, due to the non-significant direct relationship between the difficulty in verbalising emotions and the total causality of the words, mediation for this variable is not significant. 

On the other hand, total mediation was achieved for the DDF and EOT dimensions because the association of the mentioned dimensions with causality diminished to a non-significant level after introducing the mediators DDF c_2_′ = 0.01; *p* > 0.05; and EOT c_3_′ = −0.13; *p* > 0.05. Furthermore, this has been confirmed as evidenced by significant indirect effects based on the bootstrapping method with 10,000 samplings of DDF (b = 0.13, 95% CI [−0.19; −0.07]) and EOT (b = 0.07, 95% CI [−0.13; −0.03]).

### 7.3. Discussion of Results

The purpose of the analyses was to investigate to what extent the particular dimensions of alexithymia may be treated as a coherent construct in understanding its relationship with the creation of a narrative of an emotionally important experience and to what extent the influence of individual aspects varies. Therefore, in the mediation analyses presented here, each factor was verified in the models separately. As a result of the mediation analyses, the differentiated role of individual components of alexithymia in the process of narratization of a romantic relationship was demonstrated. 

#### 7.3.1. Alexithymia—Fear of Intimacy and Expressions of Negative Emotions

As is evident from the mediation model described in Figure 1, a direct effect of each alexithymia dimension on the total number of words relating to negative emotions is noticeable. However, the strongest indirect effect of influence is seen with the externally oriented thinking and fear of intimacy. Focusing on external aspects of the relationship and avoiding intimacy fosters experiencing negative emotions in the relationship and verbalising them in narratives. Externally oriented thinking to the highest degree refers to analytical, specific thinking deprived of the ability to use context, and thus symbolic and metaphorical thinking. Such understanding of the romantic relationship, “stripped” of symbols and meanings, as may be seen, favours verbalization with a greater tendency to assign negative meanings.

#### 7.3.2. Alexithymia—Fear of Intimacy and Expressions of Positive Emotions

The role of alexithymia components is interesting with respect to expressions of positive emotions. As shown in Figure 2, a direct influence applies to all dimensions of alexithymia. The indirect influence is slightly different. It turns out that externally oriented thinking is significantly related to fear of intimacy, and moreover, it is the fear of intimacy that turned out to be the main factor responsible for the lower number of positive expressions regarding the described relationships. The role of externally oriented thinking is diminishing, not to say disappearing. The other two dimensions retain their direct influence. Fear of intimacy in relation to these dimensions is not a significant factor in changing the intensity of positive words. Thus, difficulties in identifying emotions and difficulties in verbalising emotions alone are adequate for the emergence of a fear of intimacy. And they are also sufficient to minimise the positive nature of the relationship when searching for terms to describe the romantic relationship. 

#### 7.3.3. Alexithymia—Fear of Intimacy and the Use of Cognitive Categories

The third model verifies the use of cognitive categories related to causality terms. Again, individual dimensions of alexithymia contributed to a different extent. Difficulty identifying emotions and externally oriented thinking showed a direct influence, and such influence was taken over by fear of intimacy in the study of alexithymia indirect influence. It is evident from the above analysis that fear of intimacy constitutes a very important factor influencing the nature of the descriptions of romantic relationships that are created. The higher the level of alexithymia and the higher the fear of intimacy, the more difficult it is to verbalise the relationship in a balanced manner. The strength of cognitive distortions resulting from problems with understanding oneself and others in a relationship is reflected in the nature of narratives with a preponderance of negative emotions. If we consider the origin of alexithymia, then, conversely, such understanding of the relationship helps to preserve an avoidance-based attachment, i.e., one that the subjects grew up in and know well. Avoidance in a relationship is sustained by a fear of intimacy. Conversely, such a state sustains negative beliefs about others and essentially aggravates alexithymia intensity. As the analysis shows, externally oriented thinking plays a key role in this process. 

### 7.4. General Discussion

The multilevel statistical analysis that was conducted fully confirmed the adopted hypotheses. For people with a high level of alexithymia, writing a narrative about a very important aspect of life, such as a romantic relationship, turned out to be a cognitively and thus linguistically very difficult task. Looking at the results obtained, attention may be drawn to several important issues. Narratives presented by persons characterized by a high level of alexithymia are limited in generally used words and, moreover, predominant in the descriptions of romantic relationships are negative emotion words. How can such findings be interpreted? The observed fear of intimacy so strongly associated with alexithymia also informs us about undeveloped mechanisms of emotion regulation and the absence of flexibility in developing the depth of the relationship. Then, when the fear of intimacy dominates and the relationship begins to deepen, the only thing one can do to ‘protect’ themselves is to break off. Earlier, it was pointed out that the dominance of negative emotions, which are suppressed at the same time, gives protection against getting too closely involved in the relationship. For this reason, such people are not very willing to experience joy and other positive emotions because this would be a signal of intimacy and commitment to the relationship. Thus, the described narrative language of people showing high levels of alexithymia fully reflects the manner in which they construct interpersonal reality. In a sense, such structures may have a protective function against disappointment in the relationship. However, distortions that result from such narratizations may not only give rise to conflicts but also contribute to exacerbating negative effects on cognitive and affective functioning in the social environment. The specific structure of relationships consolidated in childhood is repeatedly duplicated in subsequent stages of social life. 

The results obtained may also be referred to a broader discussion on the subject of narrativity, i.e., complete, conducive to health narratives, or such that are indicative of various problems in mental functioning. Dimaggio et al. [32,33], quoted above, draw attention to impoverished narratives, where one of the subcategories includes alexithymic narratives described in detail in the theoretical section. Their essence is the absence of references to affective states, excessive focus on specifics, failure to take the recipient’s perspective, and disregard for one’s own causality. In such narratives, it is extremely difficult to distinguish between the clarity of the thread and the intention of a character. The message is vague, and, as Dimaggio et al. [32,33] indicate, such stories are “empty” when it comes to insight, emotions, or the creation of imagined sequences of events resulting from intentions, meanings, or emotions. Such narratives may hinder one’s ability to step outside of rigid and often dysfunctional patterns and foster the persistence of a negative image of oneself and one’s surroundings. It may also be associated with a lowered mood or depression [45]. Below are three examples of narratives written by people with high levels of alexithymia and fear of intimacy:

*1. “The relationship started during the pandemic, my partner had a very bad time in her life (episodes of depression). I supported her as much as I could, I felt that somehow my presence was helping her. We started to be together, her condition did not improve significantly, and she was constantly and still is depressed. I thought and told myself that when things got better with her they would get better with us too—now I see that this is not the case and it will not be. I feel tired and at the same time I love her very much and can’t imagine living without her. I just want things to be good between us, but we can’t get along or talk to each other, and she is incredibly demanding of me. I don’t feel appreciated. I’m afraid that our entire relationship is going to collapse”.*

*2. “The stages of my relationship were actually blurred. There weren’t any clear ones. Everything happened fast, there was intellectual and sexual attraction, and curiosity (very soon after the previous relationship ended). We talked a lot, we matched as we had the same priorities relating to what we wanted to achieve in the forthcoming future. Everything was going well, no surprises, no big feelings or emotions. The children were born, and it is still quite stable. But somehow there is no flair, no talk about problems. Maybe this will change when we have more time”.*

*3. “The beginning was very romantic, full of examples of soliciting me. During the relationship, the translation of my partner’s attention from myself to himself and attempts to conform to his desires, thoughts, concepts. A lot of undermining me, disregarding me and my hope that this could change. Getting into a routine, working out an ‘instruction manual’ for the relationship to somehow make it work, with a desire to save myself and my own beliefs. The ‘façade’ relationship—everything is ok from the outside, we are a compatible couple while inside there is mutual discord, alienation and a longing for things to be different”.*

The above-quoted sample narratives clearly show deficits resulting from alexithymia and relating to functioning in romantic relationships, as described extensively in the mentioned literature. Inability to understand one’s own and partner’s feelings, problems with communicating one’s own emotional needs, deficits in empathy, and thus the absence of emotional support—all these constitute convenient grounds for dissatisfaction, closing up, and conflicts. Subjects with high levels of alexithymia were motivated to avoid introspection and re-experience negative emotions emerging in their personal narratives. As is evident, there is excessive generalisation of one’s autobiographical memories (e.g., “*The stages of my relationship were actually blurred*”). Also evident is avoidance at the narrative level, such that negative events are insufficiently elaborated and inconsistent (e.g., “*I feel tired and at the same time I love her very much and can’t imagine living without her […] I’m afraid that our entire relationship is going to collapse*”). In the narratives, there are more words of negative valence and fewer positive expressions. In addition, evident is a focus on external aspects of the events described, which is not conducive to mentalizing the self in a relationship and what a partner in a relationship might think and feel. Difficulties in verbalising emotions and externally oriented thinking, supported by a fear of intimacy, foster a very specific autobiographical memory. Memories structure specific beliefs and further influence perceptions of oneself and others in the course of subsequent events. Conclusions conveyed in self-narratives such: “*I don’t feel appreciated […] she is incredibly demanding of me*” indicate generally negative beliefs about oneself and the partner, as well as misreading of others’ emotions [46]. This may lead to inadequate reactions, rejection, and resulting cautiousness when it comes to emotional intimacy. Noteworthy is also an impersonal form of communication, which indicates dissociation from emotions [4]. This is more like referring to something than writing about oneself in a relationship. This is evident in narrative 2: “*Everything was going well, no surprises, no big feelings or emotions. […] But somehow there is no flair, no talk about problems. Maybe this will change when we have more time.*” and in narrative 3: “*Getting into a routine, working out an ‘instruction manual’ for the relationship to somehow make it work, with a desire to save myself and my own beliefs*”. This breaking up of the stream of consciousness is conducive to defragmentation of the self and is present in cases of weak differentiation of the self, further exacerbating it [24].

To summarise the analysis of the survey results, we would like to indicate the role of externally oriented thinking in describing a romantic relationship. This is the result that we were positively surprised by since our previous studies showed the key role of difficulties in identifying emotions and verbalising them. Those studies explored the role of alexithymia in sustaining symptoms of alcohol dependence [4].

In this study, it was found that a direct and indirect effect of externally oriented thinking on the number of words generated in general is significant, and this is especially true for positive emotion-related words. The higher the level of externally oriented thinking and the higher the fear of intimacy, the fewer positive words were observed in the narratives (Figure 1). All alexithymia factors significantly, directly and indirectly, with fear of intimacy influenced the number of negative emotion words. This asymmetry with additionally low insight (Figure 3) unfortunately shows how distorted and defensive the understanding of one’s own romantic experience is. This raises a question: do these individuals experience less emotion in their lives, or does their deficit in processing and storing emotions decrease positive affect in their autobiographical narratives, thereby elevating a negative one? A number of researchers point to the reduced satisfaction in their relationships found in alexithymic individuals [8,26,47]. In a recent study, Lyvers et al. [9] verified satisfaction in a relationship in the context of fear of intimacy, demonstrating a negative impact on satisfaction in a relationship. In the introduction, we have discussed alexithymia and its implications for emotional development and differentiation of the self. Emotional neglect and other forms of aversive experiences in early childhood, resulting from non-secure attachment, result in impediments and deficits in emotional development [48]. Experienced rejections and interpersonal difficulties in understanding and communicating with others may further foster a fear of emotional intimacy, which hinders the development of intimate bonds in partner relationships, as was reflected in the narratives analysed.

The study conducted, despite being carried out online, dealt with romantic relationships that function in the real world. In the context of this research, we would like to refer to very interesting reports on romantic relations initiated and pursued online, and in particular to so-called cheating in romantic relationships. The intentions and motivations of people who decide to carry out this type of relationship vary considerably. We will address certain aspects of such relationships based on a meta-analysis carried out by Lazarus et al. [49]. The analysis that compares real-life relationships and online relationships described in the meta-analysis indicates several key aspects, i.e., (1) dehumanisation of partners in online relationships from the very beginning, (2) instrumental, most frequently financial, motivation to seek online relationships, also appearing as the main, and unfortunately, the only one in the case of such relationships, and (3) manipulation, including extortion or other law-breaking methods, to maximise financial and psychological exploitation of a person trapped under the pretext of a romantic relationship, evident from the very beginning in relationships aimed at romantic deception. Bombarding with love or seduction by putting oneself in the role of a person who is seeking exceptional love and is endowed with extraordinary features may in fact also take place in real-life relationships. Similarly, putting oneself in the role of a victim who finds themselves in an extremely difficult situation and needs care and support. Here, however, the probability of being discovered is definitely higher, even due to the fact that one may consult others, seek social support, etc. It is more difficult than in the virtual world to create reality, that is, unsupported by facts. Reading the meta-analysis raises the question of what contextual situations are conducive to the victim and perpetrator meeting in the virtual world. Is the hunger for bonding and relationships in a victim so strong that they are unable to recognise that they are being subjected to sophisticated manipulation? On the other hand, does a perpetrator belong to the category of people who dehumanise others in order to “hunt” for victims in a sophisticated way? Does being anonymous exempt one from basic ethics? Essential in online romantic relationships, according to the quoted meta-analysis, is gaining certain material benefits, while the methods of achieving this goal depend on developing exchanges at different types of available portals, including dating sites. All techniques and tricks applied by perpetrators of romantic-relationships-based cheating are aimed at maximising benefits. As is evident from the studies quoted, both women and men “hunt” for partners online. It is apparent that the sophisticated psychology of lying instrumentally uses language from the psychology of attachment and love for a specific, predetermined purpose. The results of our study show convincingly that real romantic relationships do have being in a relationship as their main motivation, even if it generates anxiety or difficulties. Important is the longing for attachment, no matter whether we are able to pursue it successfully. In terms of our study, it is worth posing a question about the language used by both sides of such a pseudo-romantic relationship. However, we will only be able to answer this question after we carry out another study comparing pure online relationships with those carried out in the real world.

## 8. Final Conclusions and Implications for Further Studies

The results of our study may have clinical implications. Therapy for individuals with a high level of alexithymia is extremely difficult, as psychoanalysts found out in the 1970s [1]. Externally oriented thinking is not conducive to the development of mentalization, and thus it is difficult to “reach” such people. In our next project, we will carry out a quasi-experimental study in which we intend to incorporate narrative behaviour control and teach patients new ways to narrate social reality. Externally oriented thinking is conducive to paradigmatic processing [50]. Such thinking is analytical, deprived of context, and thereby impoverishes or distorts inference or meaning attribution, not to mention mentalization. It does not trigger abstract levels of analysis based on symbols and metaphors, among others. The transition to narrative thinking is a growth step that may enhance a different perception of reality. We would like to invite couples to participate in the study, including those who are experiencing a crisis in their relationships, and through a change in narratization, they will have an opportunity to develop cognitive restructuring of difficult situations.

## Figures and Tables

**Table 1:** Descriptive statistics and Pearson correlations for study variables.

	M	SD	MIN	MAX	2	3	4	5	6	7	8	9	10
1 Age	29.14	9.17	18	75	−.132 *	−.218 **	−.079	.014	.031	−.146 *	.05	−.135 *	.003
2 TAS Toronto Alexithymia Scale	43.74	12.15	20	82		.87	.862 **	.697 **	.537 **	−.141 *	−.254 **	.366 **	.031
3 TAS DIF	16.50	6.04	7	32			.668 **	.352 **	.504**	−.065	−.212 *	.319 **	.022
4 TAS DDF	11.40	4.51	4	24				.447 **	.473 **	−.136 *	−.255 *	.295 *	.026
5 TAS EOT	15.84	4.33	8	32					.327 **	−.163 *	−.152 *	.278 *	.028
6 FIS Fear of Intimacy Scale	75.05	21.92	35	133						−.14	−.17	.327 **	−.011
7 LIWC Word count	133.50	152.02	1	1604							−.113	.059	.099
8 LIWC Positive emotions	4.28	3.50	0	25								−.213 **	−.147 *
9 LIWC Negative emotions	1.68	1.61	0	7.84									.073
10 LIWC Cause	1.42	1.76	0	15.38									1

TAS—Toronto Alexithymia Scale; DIF—difficulty identifying feelings; DDF—difficulty describing feelings; EOT—externally oriented thinking; FIS—Fear of Intimacy Scale; LIWC—Linguistic Inquiry Word Count. * *p* < 0.05; ** *p* < 0.01.

**Table 2:** One-way analyses of variance for a group of subjects who meet the criteria for alexithymia or probable alexithymia (more than 51 points) compared to those who are non-alexithymic.

	Groups					
	Non Alexithymic		Alexithymic		F	η^2^
	M	SD	M	SD		
LIWC Word count	144.38	133.30	87.56	70.96	12.536 ***	.07
LIWC Positive emotions	5.18	3.03	3.22	3.05	18.429 ***	.09
LIWC Negative emotions	1.21	1.02	2.55	1.82	33.367 ***	.16
LIWC Cause	2.25	1.91	1.19	1.04	6.071 *	.03
FIS Fear of intimacy	63.98	16.95	91.38	21.13	68.372 ***	.34

* *p* < 0.05; *** *p* < 0.001.

## Data Availability

All authors gave consent for full data access.
